# Effect of Stressors on the mRNA Expressions of Neurosecretory Protein GL and Neurosecretory Protein GM in Chicks

**DOI:** 10.3389/fphys.2022.860912

**Published:** 2022-03-15

**Authors:** Masaki Kato, Eiko Iwakoshi-Ukena, Yuki Narimatsu, Megumi Furumitsu, Kazuyoshi Ukena

**Affiliations:** Laboratory of Neurometabolism, Graduate School of Integrated Sciences for Life, Hiroshima University, Higashi-Hiroshima, Japan

**Keywords:** neurosecretory protein, chicken, hypothalamus, stress, histamine

## Abstract

We recently discovered novel cDNAs encoding the precursors of two small secretory proteins, neurosecretory protein GL (NPGL) and neurosecretory protein GM (NPGM), in the mediobasal hypothalamus (MBH) of chickens. In addition, we found colocalization of NPGL, NPGM, and histidine decarboxylase (HDC; histamine-producing enzyme) in same neurons of the medial mammillary nucleus of the hypothalamus. In this study, we elucidated the effect of several stresses, including food deprivation, environmental heat, inflammation, and social isolation, on the mRNA expression of *NPGL*, *NPGM*, and *HDC* in chicks using real-time PCR. Food deprivation for 24 h increased *NPGM* mRNA expression in the MBH. On the other hand, an environmental temperature of 37°C for 24 h did not affect their mRNA expression. Six hours after intraperitoneal injection of lipopolysaccharide, an inducer of inflammation, the mRNA expression of *NPGM*, but not that of *NPGL* and *HDC* increased. Social isolation for 3 h induced an increase in the mRNA expression of *NPGL*, *NPGM*, and *HDC*. These results indicate that NPGM, but not NPGL or HDC, may participate in several physiological responses to stress in chicks.

## Introduction

Biological stress responses are induced through the hypothalamus, which is known as the central region of the autonomic nervous system and endocrine system. In particular, the hypothalamic-pituitary-adrenal (HPA) axis is the most predominant pathway for the endocrine response to stress in vertebrates (Tsigos and Chrousos, 2002). In birds, exposure to stress induces the hypothalamus to secrete neuropeptides, corticotropin-releasing factor (CRF), and arginine vasotocin (AVT) in the paraventricular nuclei (PVN); and stimulates the pituitary gland to release adrenocorticotropic hormone (ACTH; Barth and Grossmann, 2000; Jenkins and Porter, 2004). ACTH, secreted by the pituitary gland, stimulates the adrenal cortex through the bloodstream causing the synthesis and secretion of adrenal cortex hormones. Corticosterone is another biological marker of stress in birds (Dmitriev et al., 2001; Feltenstein et al., 2003; Pereyra and Wingfield, 2003). As chickens are one of the most important food resources, research on stress responses affecting egg and meat production is important (Tan et al., 2010; Mignon-Grasteau et al., 2015; Nawaz et al., 2021). It is crucial to understand how stress response is regulated in the brain in order to minimize the effects of stress on the whole body. However, all pathways responsible for stress response in the chicken brain remain unknown. Therefore, novel factors involved in the stress response need to be investigated.

Recently, we discovered two novel cDNAs, paralogous genes, that encode the precursors of small secretory proteins in the mediobasal hypothalamus (MBH) of chickens (Ukena et al., 2014; Ukena, 2018). Because the predicted C-terminal amino acids of the small protein were Gly-Leu-NH_2_ and Gly-Met-NH_2_, the two small secretory proteins were named neurosecretory protein GL (NPGL) and neurosecretory protein GM (NPGM), respectively. Genomic database analysis showed that homologous genes of these novel cDNAs are highly conserved in vertebrates (Ukena, 2018). In chicks, NPGL and NPGM are produced in the medial mammillary nucleus (MM) and the infundibular nucleus (IN) of the hypothalamus (Shikano et al., 2018a). In particular, NPGL and NPGM are co-localized in the same neurons of the MM (Shikano et al., 2018a). We found that NPGL induces hyperphagia and fat deposition in rodents and chicks (Iwakoshi-Ukena et al., 2017; Shikano et al., 2018b, 2019; Fukumura et al., 2021). In contrast, NPGM induces fat deposition without hyperphagia in male chicks (Kato et al., 2021). Our previous studies suggest that NPGL and NPGM are related to energy homeostasis and/or growth in the hypothalamus of vertebrates.

Food deprivation is also a known naturally occurring stressor (Méndez and Wieser, 1993; Midwood et al., 2016). Our previous studies showed that *NPGL* mRNA expression levels were elevated by food deprivation in mice and rats (Iwakoshi-Ukena et al., 2017; Matsuura et al., 2017). In addition, we found the colocalization of NPGL, NPGM, and histidine decarboxylase (HDC; histamine-producing enzyme) in the MM of the chick hypothalamus (Bessho et al., 2014; Shikano et al., 2018a). HDC is a rate-limiting enzyme that synthesizes histamine from histidine. Neuronal histamine has a variety of physiological functions as a neurotransmitter or neuromodulator in the mammalian brain (Ito, 2000). In rodents, neuronal histamine is known to be involved in stress response. Histamine turnover is increased by various stressful situations in the brain, especially in the hypothalamus (Itoh et al., 1989; Ito, 2000). These results suggest that NPGL and NPGM play important roles in stress responses in rodents. There are many different types of stresses, such as energy deficiency, ambient temperature, inflammation, and social isolation. However, it is unclear whether NPGL, NPGM, and HDC respond to these stresses in birds. In this study, we analyzed the mRNA expression of *NPGL*, *NPGM*, and *HDC* in chickens exposed to different types of stressors.

## Materials and Methods

### Animals

Male layer chicks of 1-day-old were purchased from a commercial company (Nihon layer, Gifu, Japan) and housed in groups in a windowless room at 28°C on a 20-h light (4:00–24:00)/4-h dark (0:00–4:00) cycle. Except for those in the food deprivation experiment, all chicks had *ad libitum* access to food and water.

### 24-h Food Deprivation Experiment

Male layer chicks (*n* = 6) of 17-day-old were fasted for 24 h. The chicks had *ad libitum* access to water. The control chicks (*n* = 5) had *ad libitum* access to food and water. Every 12 h, blood samples were collected from the ulnar veins. Blood glucose levels were measured using a GLUCOCARD G+ meter (Arkray, Kyoto, Japan). After fasting, the chicks were euthanized, and the MBH was collected. The MBH which includes the MM, IN, ventromedial hypothalamus, and periventricular stratum was dissected out using fine forceps and small scissors (Shikano et al., 2018b). The MBH was immediately snap-frozen in liquid nitrogen and stored at −80°C for real-time PCR analysis.

### Heat Stress Experiment

Male layer chicks (*n* = 6) of 14-day-old were housed at 37°C for 24 h. The control chicks (*n* = 6) were housed at 25°C. After the experiment, the chicks were euthanized, and the MBH was collected for real-time PCR analysis. Blood samples were collected in heparinized tubes at the endpoint.

### Lipopolysaccharide-Induced Inflammation Experiment

Male layer chicks (*n* = 7) of 6-day-old were administered intraperitoneal (i.p.) injection of 2 mg/kg (body mass) lipopolysaccharide (LPS) from *Escherichia coli* O127 (Wako Pure Chemical Industries, Osaka, Japan). LPS was dissolved in 0.9% NaCl. The control chicks (*n* = 5) were treated with an i.p. injection of 0.9% NaCl. After 6 h, the chicks were euthanized and the MBH was collected for real-time PCR analysis. Blood samples were collected at the endpoint.

### Social Isolation Stress Experiment

Male layer chicks were housed in groups until 7-day-old and had *ad libitum* access to food and water. The chicks (*n* = 5) were then isolated from the group breeding and housed individually for 3 h. The control chicks (*n* = 6) were housed in groups. After isolation, the chicks were euthanized and the MBH was collected for real-time PCR analysis. Blood samples were collected at the endpoint.

### Blood Corticosterone Test

Blood samples were centrifuged at 800 × *g* for 15 min at 4°C to separate the blood plasma. Blood plasma samples were used to measure the corticosterone levels by a commercial ELISA kit (Assaypro, St. Charles, MO, United States).

### Real-Time PCR

RNA was extracted using RNAqueous-Micro Kit (Life Technologies, Carlsbad, CA, United States) following the manufacturer’s instructions. First-strand cDNA was synthesized from total RNA using ReverTra Ace qPCR RT Master Mix with gDNA Remover (TOYOBO, Osaka, Japan). PCR amplification was performed using THUNDERBIRD SYBR qPCR Mix (TOYOBO): 95°C for 20 s followed by 40 cycles at 95°C for 3 s and 60°C for 30 s using a Step One Real-time Thermal Cycler (Applied Biosystems, Bedford, MA, United States). The ROX reference dye was added into each well to normalize the fluorescence intensity of SYBR green in real-time PCR. Relative quantification for each expression was determined by the 2^−ΔΔ*C*t^ method using β-actin (*ACTB*) as an internal control. The primer sequences for *NPGL*, *NPGM*, *HDC*, and the gene of neuropeptide Y (*NPY*) are listed in Supplementary Table 1. *NPY* is frequently used as a biological marker for fasting experiments in chickens (Boswell et al., 1999).

### Statistical Analysis

Data were analyzed using the Student’s *t*-test or one-way ANOVA for mRNA expression, blood glucose levels, and corticosterone levels in blood plasma. The significance level was set at *p* < 0.05. All results are expressed as the mean ± SEM.

## Results

### Response to the 24-h Food Deprivation

**Figure 1:**
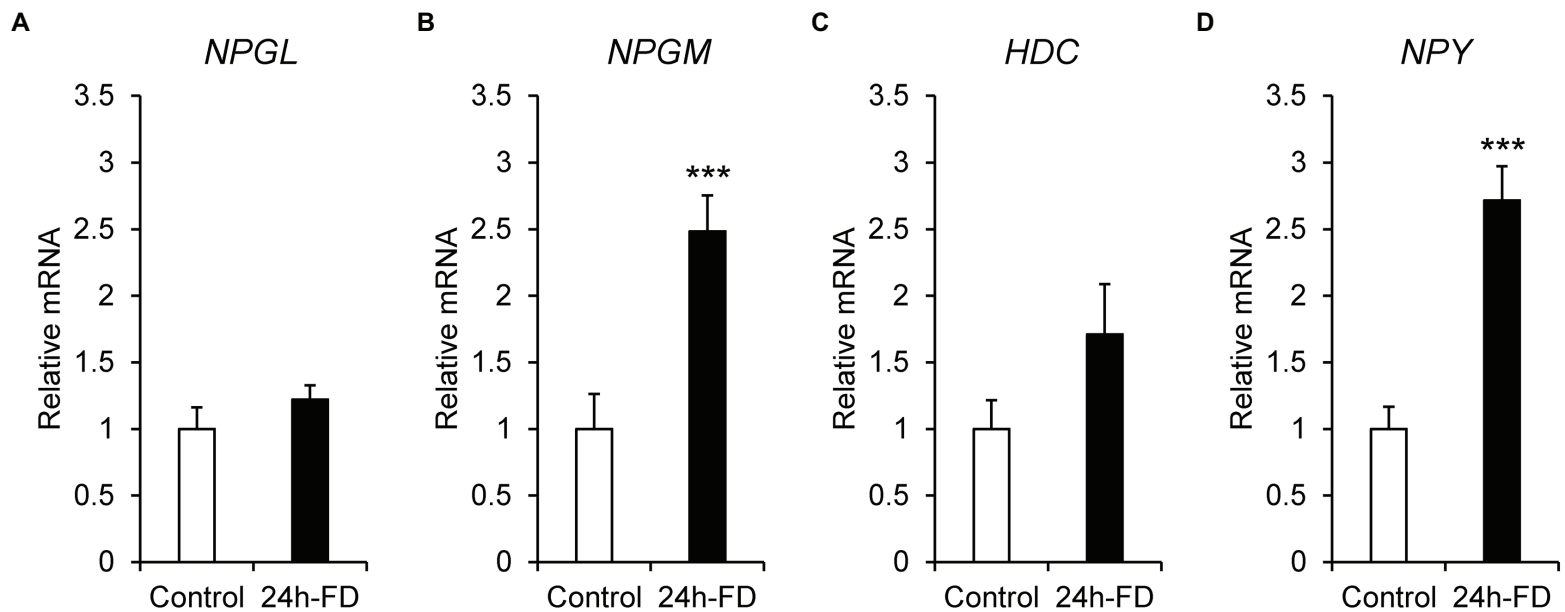
mRNA expression levels, neurosecretory protein GL (*NPGL*; **A**), neurosecretory protein GM (*NPGM*; **B**), histidine decarboxylase (*HDC*; **C**), neuropeptide Y (*NPY*; **D**) in the mediobasal hypothalamus in the 24 h food deprivation experiment. Data are expressed as the mean ± SEM (*n* = 5–6). Data were analyzed by Student’s *t*-test. An asterisk indicates a statistically significant difference (^***^p < 0.005).

To investigate the stress response of NPGL, NPGM, and HDC to 24 h food deprivation, we analyzed the mRNA expression of *NPGL*, *NPGM*, *HDC*, and *NPY*. The results showed that the mRNA expression of *NPGM* (*p* < 0.005, *F* = 1.26) and *NPY* (*p* < 0.005, *F* = 2.87) significantly increased after 24 h fasting (Figures 1B,D). However, *NPGL* (*p* = 0.27, *F* = 1.86) and *HDC* (*p* = 0.16, *F* = 3.65) did not change (Figures 1A,C). In this experiment, blood glucose levels (*p* < 0.005, *F* = 12.99) decreased after 24 h of food deprivation (Supplementary Figure 1).

### Response to Heat Stress

**Figure 2:**
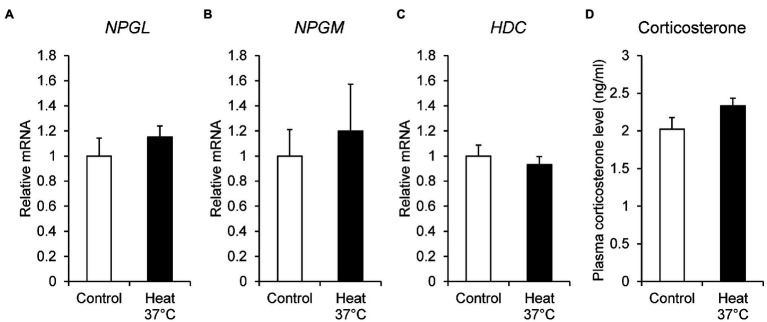
mRNA expression levels, neurosecretory protein GL (*NPGL*; **A**), neurosecretory protein GM (*NPGM*; **B**), histidine decarboxylase (*HDC*; **C**) in the mediobasal hypothalamus in the heat stress experiment at 37°C for 24 h. Plasma corticosterone concentration level **(D)**. Data are expressed as the mean ± SEM (*n* = 6). Data were analyzed by Student’s *t*-test.

To investigate the effect of ambient temperature, we examined the mRNA expression of *NPGL*, *NPGM*, and *HDC* under heat stress. The chicks were kept at 37°C for 24 h, and then the mRNA expression levels of *NPGL*, *NPGM*, and *HDC* were quantified by real-time PCR. Corticosterone concentration was determined as a biological marker of stress in birds. The *NPGL* (*p* = 0.37, *F* = 0.44), *NPGM* (*p* = 0.65, *F* = 3.17), and *HDC* (*p* = 0.53, *F* = 1.56) mRNA expression levels (Figures 2A–C), and plasma corticosterone concentration (*p* = 0.12, *F* = 0.43; Figure 2D) were not affected by heat stress.

### Response to LPS-Induced Inflammation

**Figure 3:**
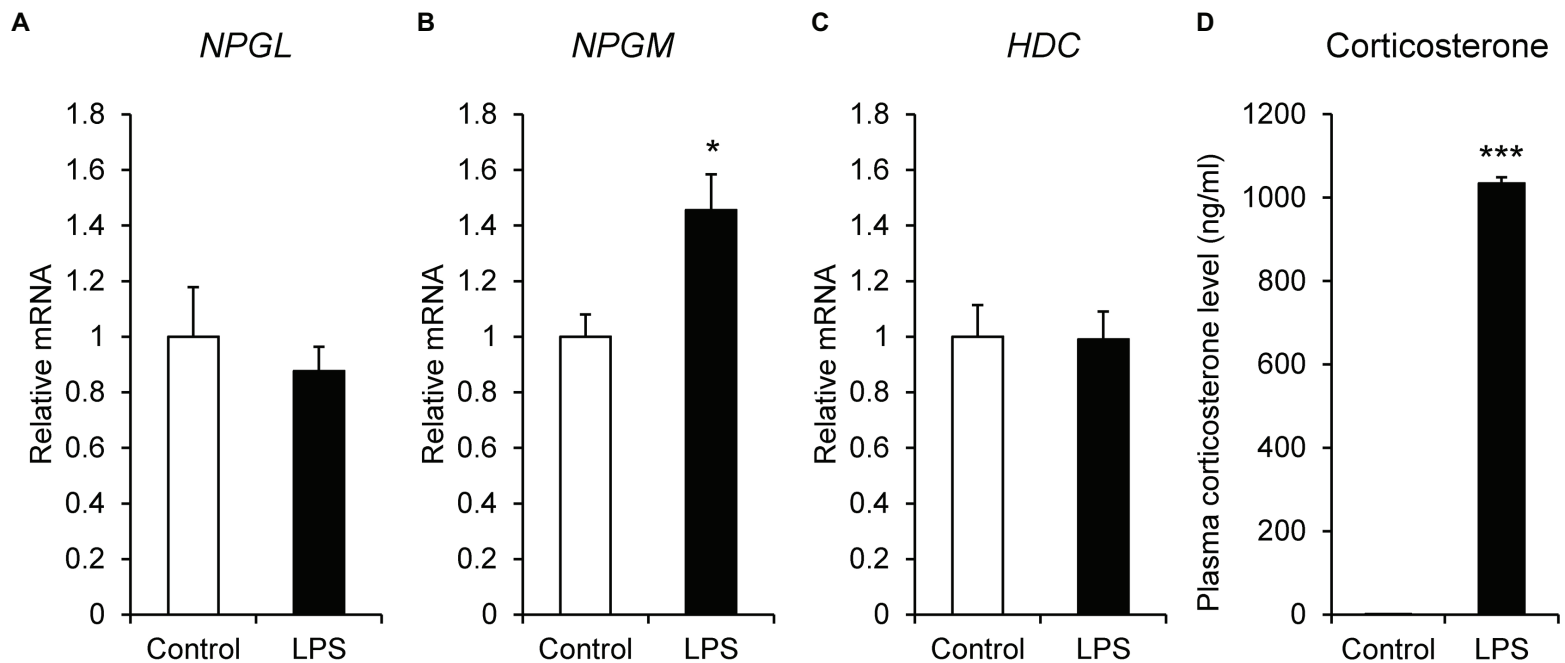
mRNA expression levels, neurosecretory protein GL (*NPGL*; **A**), neurosecretory protein GM (*NPGM*; **B**), histidine decarboxylase (*HDC*; **C**) in the mediobasal hypothalamus in the lipopolysaccharide (LPS)-induced inflammation stress experiment. Plasma corticosterone concentration level **(D)**. Data are expressed as the mean ± SEM (*n* = 5–7). Data were analyzed by Student’s *t*-test. An asterisk indicates a statistically significant difference (^*^p < 0.05, ^***^p < 0.005).

LPS is an endotoxin derived from the outer membrane of gram-negative bacteria and is used to induce inflammatory stress. Thus, we administered i.p. injection of LPS to the chicks and quantified the mRNA expression levels of *NPGL*, *NPGM*, and *HDC* by real-time PCR. The mRNA expression of *NPGM* (*p* < 0.05, *F* = 3.57) significantly increased after i.p. injection of LPS (Figure 3B). However, the expression levels of *NPGL* (*p* = 0.51, *F* = 2.90) and *HDC* (*p* = 0.95, *F* = 0.92) mRNA did not change (Figures 3A,C). In addition, plasma corticosterone concentration (*p* < 0.005, *F* = 0.00029) significantly increased after the i.p. injection of LPS (Figure 3D).

### Response to Social Isolation Stress

**Figure 4:**
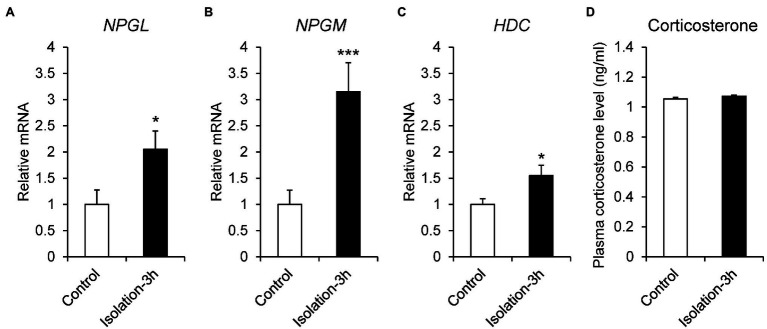
mRNA expression levels, neurosecretory protein GL (*NPGL*; **A**), neurosecretory protein GM (*NPGM*; **B**), histidine decarboxylase (*HDC*; **C**) in the mediobasal hypothalamus in the isolation stress experiment for 3 h. Plasma corticosterone concentration level **(D)**. Data are expressed as the mean ± SEM (*n* = 5–6). Data were analyzed by Student’s *t*-test. An asterisk indicates a statistically significant difference (^*^p < 0.05, ^***^p < 0.005).

Social isolation is a strong stressor, because chicks spend time in a group after hatching. To investigate the stress response to social isolation stress, 7-day-old chicks were isolated for 3 h, and the mRNA expression of *NPGL*, *NPGM*, and *HDC* was quantitated by real-time PCR. The results showed that the expression levels of *NPGL* (*p* < 0.05, *F* = 1.33), *NPGM* (*p* < 0.005, *F* = 3.37), and *HDC* (*p* < 0.05, *F* = 2.67) mRNA significantly increased after the isolation (Figures 4A–C). The isolated chicks were keeping to make distress calls for 3 h (data not shown), although plasma corticosterone concentrations (*p* = 0.21, *F* = 0.33) did not change after the isolation (Figure 4D).

## Discussion

This study is the first to investigate the effect of various stress conditions on the mRNA expression of *NPGL*, *NPGM*, and *HDC* in the MBH. We selected four stressors: food deprivation, heat stress, inflammation stress, and isolation stress. The results of the stress experiments suggested that the changes in the mRNA expressions of *NPGL*, *NPGM*, and *HDC* depend on the type of stressor. *NPGM* mRNA expression was upregulated by food deprivation, inflammation stress, and social isolation stress. In contrast, the mRNA expression of *NPGL* and *HDC* was only increased by social isolation stress, although they were co-expressed with NPGM in the same neurons of the chicken MM (Shikano et al., 2018a). Heat stress at 37°C for 24 h had no effect on the mRNA expression of these genes. These results suggest that NPGM is more sensitive to energy deficiency, inflammatory response, and isolated states than NPGL or HDC.

Food deprivation experiments are widely used to understand the regulation and molecular mechanisms of feeding in many animals. Food deprivation for 24 h increased the mRNA expression of *NPGM*, whereas that of *NPGL* and *HDC* did not change. This result shows that hunger signaling regulates only the mRNA expression of *NPGM* in NPGL/NPGM/HDC-expressing neurons. The hypothalamus in chicks receives hunger signals, such as hypoglycemia, hormones from peripheral tissues, and other regions of the brain (Richards and Proszkowiec-Weglarz, 2007). For example, the central melanocortin system is one of the most characterized neuronal pathways in the regulation of feeding behavior and energy expenditure (Cone, 2005). In addition to melanocortin, when hunger signals *via* the blood or vagus nerve are transmitted from the peripheral tissues, NPY- and agouti-related peptide (AgRP)-expressing neurons are subsequently activated, which increases feeding behavior in chicks (Richards and Proszkowiec-Weglarz, 2007). In this study, *NPY* mRNA expression was increased by food deprivation for 24 h, and blood glucose levels gradually decreased during fasting. These responses indicated that the hunger signal was being generated adequately. Recently, a part of the NPY-related hunger signal pathway had been elucidated in rat brain (Nakamura et al., 2017). It has been reported that NPY, during hunger, activates GABAergic neurons in the reticular nuclei of the medulla oblongata, and GABAergic neurons inhibit energy expenditure (Nakamura et al., 2017). Our previous study showed that NPGM induces fat accumulation without increasing feeding behavior in chicks (Kato et al., 2021). It is possible that NPGM activated by hunger signaling may inhibit energy expenditure *via* GABAergic neurons. The relationship between NPGM-expressing neurons and GABAergic neurons in chicks is still unclear. Therefore, further studies are necessary to elucidate the biological significance of NPGM during the hunger period.

It is well known that heat stress is a particularly important environmental stressor, as it changes physiological responses, reduces the ability to digest nutrients, and decreases growth rate in chicks (Lara and Rostagno, 2013; Lu et al., 2017; Chowdhury, 2019). In addition, heat stress is often associated with high mortality in chicks. Exposure of chickens to high temperatures activates the HPA axis *via* the PVN from the preoptic area and leads to secretion of corticosterone (Bohler et al., 2021). Heat stress inhibits the mRNA expression of *NPY* and induces anorexia (Bohler et al., 2020). In mice, parts of CRF neurons from the PVN project into NPY neurons and inhibit these neurons (Stengel et al., 2009; Kuperman et al., 2016). The same phenomenon may occur in birds. However, the relationship between CRF neurons and NPGL/NPGM/HDC-expressing neurons remains unclear. In this study, heat stress at 37°C for 24 h did not change the mRNA expression of *NPGL*, *NPGM*, and *HDC* in the MBH. Additionally, the blood plasma corticosterone levels did not change, suggesting that it was stable on the HPA axis during this period. In chicks, corticosterone concentration is known to increase in response to acute (12 h) heat stress, but subsequently decreases to normal levels after heat stress for 24 h (Furukawa et al., 2016). The present data was coincident with the previous study (Furukawa et al., 2016). Therefore, it is possible that the mRNA expression of *NPGL*, *NPGM* and *HDC* may be affected by heat stress for 12 h. Future studies should investigate the effects of short-time heat stress on the mRNA expression of *NPGL*, *NPGM*, and *HDC* in chickens.

Injection of LPS has often been employed to study systemic inflammatory reactions (Tonelli et al., 2008). LPS activates macrophagic response and induces oxidative stress in a dose-dependent manner (Koumakis et al., 2019). LPS-induced inflammation is also used in chicks, similar to that in mammals (Zhang et al., 2013; Liu et al., 2020). The i.p. injection of LPS induces hyperthermia and anorexia and causes inflammation stress and tissue damage in chicks (Johnson et al., 1993). LPS-induced inflammation is known to induce the mRNA expression of cytokines such as interleukin-1β (*IL-1β*) and interleukin-6 (*IL-6*) in chicken peripheral tissues and hypothalamus (Zhang et al., 2013; Grabbe et al., 2020). In particular, IL-6 induces calcium signaling in the hypothalamic neurons of chickens (Grabbe et al., 2020). Inflammation and immunoreactivity lead to energy wastage, thus, inhibiting the growth in chicks (Takahashi et al., 2008). In this study, plasma corticosterone levels were drastically increased by LPS-induced inflammation. Since other studies have shown that the same treatment increases corticosterone levels (Johnson et al., 1993), the inflammatory response is correctly induced. LPS-induced inflammation increased *NPGM* mRNA expression in the MBH. This result suggests that *NPGM* mRNA expression in NPGL/NPGM/HDC-expressing neurons may be activated by inflammatory cytokines. Although the pathway that promotes the transcription of *NPGM* has not been identified yet, the cytokine signaling pathway may promote the transcription of *NPGM*. In the future, it will be necessary to understand the relationship between NPGM and cytokines, such as IL-1β and IL-6.

It has been reported that social isolation stress can change the reactivity to stress and social behavior in both animals and humans, and it has been used as a biobehavioral assay to study attachment processes (Panksepp et al., 1980; Sufka et al., 1994). Social isolation stress activates various endocrinological changes, such as the HPA axis and secretion of glucocorticoids and catecholamines (Tsigos and Chrousos, 2002). In birds, this stress is thought to be related to the growth rate (John Savory and MacLeod, 1980). It has been reported that social isolation stress activates the HPA axis and stimulates the secretion of corticosterone after 10 min of isolation in layer chicks (Saito et al., 2005). In this study, there was no change in plasma corticosterone levels after 3 h of isolation, although chicks were making distress calls for 3 h. However, the mRNA expression of *NPGL*, *NPGM*, and *HDC* increased after 3 h of isolation. It is possible that the expression of *NPGL*, *NPGM*, and *HDC* is independent of the HPA axis in this experiment. The c-Fos expression in a variety of brain regions during isolation stress has been mapped in Japanese quails (Takeuchi et al., 1996). The density of c-Fos-immunoreactive cells is much higher in stressed groups than that of non-stressed groups in the dorsomedial mesencephalic areas (DMM; Takeuchi et al., 1996). We have identified that histamine neurons are localized in the MM and projected to these areas in chickens (unpublished data). We speculate that NPGL/NPGM/HDC-expressing neurons are activated by isolation stress and that NPGL, NPGM, and histamine are transmitted to a variety of brain regions. In the future, we need to analyze the projection of NPGL/NPGM/HDC-expressing neurons throughout the chick brain, and the regulatory mechanism of mRNA expression under isolation stress.

In conclusion, the present study revealed that the expression of *NPGL*, *NPGM*, and *HDC* depends on the type of stressor and may regulate stress responses to protect the whole body from stressors. This study shows that new factors contribute to stress mechanisms in chickens. Our previous study showed that the mRNA expression levels of *NPGL* increased, and *NPGM* decreased during post-hatching development in chicks (Shikano et al., 2018a). Thus, NPGL and NPGM have different physiological functions in addition to stress responses in chicks. However, the biological significance of the changes in mRNA expression under different stressors was not obvious in the present study. Future studies on the physiological functions of these brain factors and the gene-regulatory networks within the same neuron under stress are necessary. As the promoter regions of *NPGL*, *NPGM*, and *HDC* have not been studied yet, the presence of the stress response elements (STRE; Freitas et al., 2016; Iwata et al., 2017) in these regions remains unknown. On the other hand, this data was obtained using layer chicks. The layer is slower-growing and developed for egg production, while the broiler is fast-growing and selected for meat production. It is known that layers and broilers have different physiological and behavioral responses to stressors (Khan et al., 2015). Future studies need to analyze the mRNA expression of *NPGL*, *NPGM*, and *HDC* in broilers. Chickens must be reared under stress free conditions to prevent a decline in production and animal welfare. Therefore, elucidation of the molecular mechanisms of novel brain factors, NPGL and NPGM, may help to mitigate stresses in chickens.

## Data Availability Statement

The original contributions presented in the study are included in the article/Supplementary Material, further inquiries can be directed to the corresponding author.

## Ethics Statement

The animal study was reviewed and approved by the experimental protocols were in accordance with the Guide for the Care and Use of Laboratory Animals prepared by Hiroshima University (Higashi-Hiroshima, Japan).

## Author Contributions

MK and KU: conceptualization. MK, EI-U, YN, MF, and KU: methodology and investigation. MK: writing—original draft preparation and visualization. MK, YN, and KU: writing—review and editing. KU: project administration. EI-U and KU: funding acquisition. All authors contributed to the article and approved the published version of the manuscript.

## Funding

This work was supported by JSPS KAKENHI Grant (JP19K06768 to EI-U, JP19H03258, and JP20KK0161 to KU), and the Kieikai Research Foundation (KU).

## Conflict of Interest

The authors declare that the research was conducted in the absence of any commercial or financial relationships that could be construed as a potential conflict of interest.

## Publisher’s Note

All claims expressed in this article are solely those of the authors and do not necessarily represent those of their affiliated organizations, or those of the publisher, the editors and the reviewers. Any product that may be evaluated in this article, or claim that may be made by its manufacturer, is not guaranteed or endorsed by the publisher.
