# Supplementary material for: Effect of Stressors on the mRNA Expressions of Neurosecretory Protein GL and Neurosecretory Protein GM in Chicks
Source: Front Physiol. 2022 Mar 15;13:860912. doi: 10.3389/fphys.2022.860912 (PMC8964992; doi:10.3389/fphys.2022.860912)
Supplement: Supplementary file 1 [file Data_Sheet_1.PDF]

Supplementary Material

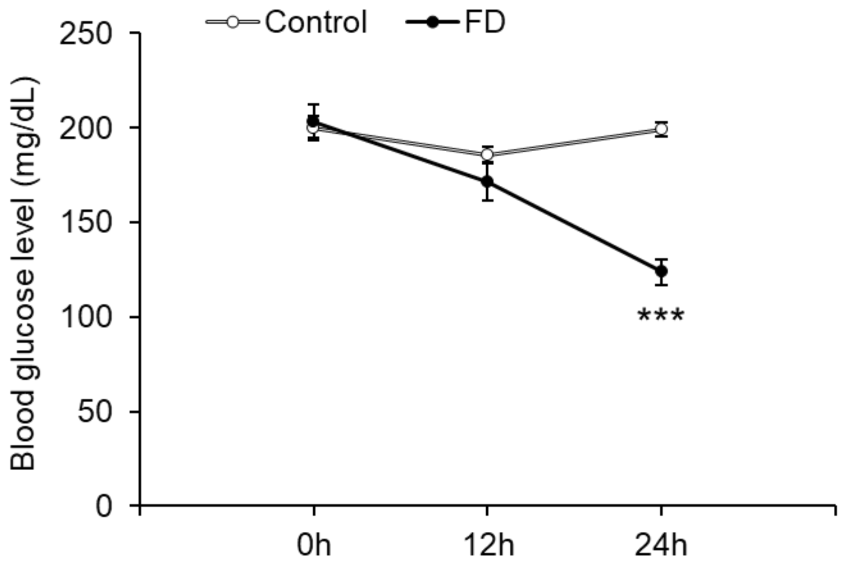

Supplementary Figure 1. Fluctuations in blood glucose levels during food depravation for 24 h. Data are expressed as the mean ± SEM (n = 5–6). Data were analyzed using one-way ANOVA. Asterisks indicate statistically significant differences (****P* < 0.005).

Supplementary Table 1. Sequences of oligonucleotide primers for real-time PCR

Gene	Forward primer	Reverse primer
NPGL	CTAGGAAAAAGACAGCTTGC	CTTTCTTCGTCAGAACTGGT
NPGM	ATGGAATTCATGTGGAAGAG	AGCATCTACAGTAAATGCTG
HDC	GTAAGAAGTGATCCGCTGTT	TGCTCAGTTCTTTCAGGAGT
NPY	ACATGGCCAGATACTACTCG	ACAAGAGGTCTGAGATCAGTG
ACTB	CCAGAGTCCATCACAATACC	AGCCAACAGAGAGAAGATGA
